# Raised Maternal Homocysteine Levels in Antenatal Women at 10 to 14 Weeks of Gestation and Placenta-Mediated Complications: A Cohort Study

**DOI:** 10.7759/cureus.40423

**Published:** 2023-06-14

**Authors:** Priyanka Thakur, Anuja Bhalerao

**Affiliations:** 1 Department of Obstetrics and Gynaecology, NKP Salve Institute of Medical Sciences and Research Centre, Nagpur, Maharashtra, IND

**Keywords:** maternal and fetal outcomes, gestational age, placenta mediated complications, antenatal women, homocysteine levels

## Abstract

Background

Placenta-mediated complications, such as preeclampsia, placental abruption, and fetal growth restriction, can indeed lead to significant maternal and perinatal morbidity and mortality. Early detection and management of these conditions are crucial to ensuring optimal outcomes for both the mother and baby. However, there have been inconsistent correlations found between maternal homocysteine levels and placenta-related problems in various studies. Therefore, prospective research based on data pointing to a role for hyperhomocysteinemia in placenta-mediated complications will open doors for early detection and management of these complications. Thus, this study aims to determine if a higher risk of placenta-mediated problems is connected with a higher maternal plasma homocysteine content between 10 and 14 weeks of gestation.

Methodology

An observational prospective cohort study was conducted in the Department of Obstetrics and Gynecology, consisting of all the antenatal women between 10 and 14 weeks of gestation attending outpatient departments or inpatients admitted in labor rooms or wards having singleton pregnancies. Along with socio-demographic information and detailed history, a clinical examination was performed, and blood samples were collected to determine plasma homocysteine levels.

Results

As per the receiver operating characteristic curve (ROC curve), the cut-off value taken was <5 for the low level of serum homocysteine, 5 to 15 micromol/L for the normal value, and >15 micromol/L for a raised serum homocysteine level. The cutoff value for our study was 45 micromol/L with a sensitivity of 78.33%, a specificity of 91.67%, a positive predictive value of 90.38%, and a negative predictive value of 80.88% with a diagnostic accuracy of 85%. This means that, for most of the women included in the present study, those who developed placenta-mediated complications had serum blood homocysteine levels of 45 micromol/L or more at 10-14 weeks of gestation.

Conclusion

Women with high homocysteine levels in the late first trimester had more placenta-mediated complications, such as abruption, pre-eclampsia, restricted fetal growth, and recurrent pregnancy losses, compared to women with a normal level of homocysteine in the late first trimester. Therefore, measuring blood homocysteine levels in pregnancy may be helpful as a diagnostic test for the early detection of high-risk individuals for placenta-mediated complications.

## Introduction

Pregnancy prepares the mother for various hemostatic challenges of delivery by creating a physiologically hypercoagulable state, resulting in a higher risk of problems caused by the placenta and venous thrombosis, which pose significant difficulties for both the mother and the fetus. These placenta-mediated issues, which can complicate up to 15% of pregnancies, must be avoided for the benefit of women's health [1].

Placenta-mediated difficulties represent 5%-15% of all pregnancy-related problems in developing countries and have an adverse effect on both the mother and the fetus. Small for gestational age (SGA), fetal growth restriction (FGR), or intrauterine growth restriction (IUGR), hypertensive diseases such as placental abruption, preeclampsia, and recurrent pregnancy loss are the main indications of impaired placental function throughout pregnancy [2]. These unfavorable maternal and fetal outcomes are primarily caused by vascular-related pregnancy problems. The problem is thought to have its foundation in the early placentation process, which includes trophoblast invasion and angiogenesis and also depends on the function of vascular and endothelial cells. According to recent studies, numerous pregnancy issues may be associated with maternal plasma homocysteine levels. A proposed theory is that hyperhomocysteinemia may be associated with certain placental complications, such as preeclampsia [3,4]. Homocysteine levels typically decrease throughout pregnancy, which may be a result of the pregnancy-related increase in glomerular filtration rate, the increase in plasma volume and associated hemodilution, and a possible increased fetal homocysteine uptake. The elevated maternal homocysteine concentrations may negatively impact placental development in the early stages of pregnancy [4].

An increased total plasma homocysteine content is one of the metabolic consequences of folate insufficiency. Based on a theory, disruption in the vascular system of the placenta is thought to be more common in women because elevated homocysteine causes damage to the endothelial cells [5], and changes in the level of homocysteine in the mother's blood result in placental vasculopathy, which causes intrauterine growth restriction (IUGR), preeclampsia, abruption, and recurrent miscarriages. These disorders share common placental pathophysiology, have a higher risk of recurrence, and have been linked to aberrant placental vasculature. In addition to fetal growth restriction, impaired placental transport, and the delivery of low-birth-weight (LBW) neonates in the current pregnancy, the effects of homocysteine on pregnancy outcomes have also been linked to other unfavorable antepartum events like oligohydramnios and meconium staining of amniotic fluid [2,6].

One of the leading causes of maternal and perinatal morbidity and mortality is placenta-mediated problems. Early detection and efficient care of these problems are thus necessary. Inconsistent correlations between maternal homocysteine, measured at various stages of pregnancy, and difficulties caused by the placenta have been found in several studies, and according to various research, there is an essential link between placenta-mediated problems and hyperhomocysteinemia [2,6-10]. Therefore, prospective research based on data pointing to a role for hyperhomocysteinemia in placenta-mediated complications will open the door for better management of pregnant women and their unborn children. Hence, this study aims to determine if a higher risk of placenta-mediated problems is connected with a higher maternal plasma homocysteine content between 10 and 14 weeks of gestation.

## Materials and methods

An observational prospective cohort study was conducted in the Department of Obstetrics and Gynecology at a tertiary care hospital from January 2021 to December 2022 after approval from the Institutional Ethical Committee (IEC) with reference number 99/2021, which consisted of all the antenatal women between 10 and 14 weeks of gestation attending outpatient departments or inpatient in labor rooms or wards having a singleton pregnancy. Whereas patients with diagnosed congenital anomalies in the fetus, previously identified fetal growth restriction, oligohydramnios, known cases of chronic hypertension, renal disease, diabetes mellitus, recurrent pregnancy losses like incompetent os, fibroid, and congenital anomalies of the uterus were excluded from the study. Based on the inclusion and exclusion criteria, a convenient sampling of 120 antenatal women was included in the present study after receiving written informed consent.

A detailed history regarding demographic, obstetric, menstrual, past, drug, personal, and family parameters was taken, followed by a per abdomen examination for the period of gestation, lie, presentation, the position of the baby, amniotic fluid, and baby weight, along with a per speculum/per vaginal examination that was performed to note cervicovaginal infection, cervical status, length of the cervix, leakage of liquor, cervical effacement, and dilatation.

All antenatal routine investigations were done, and a 2cc blood sample for homocysteine was collected in EDTA vacutainer tubes and transported to the laboratory within 30min. Plasma homocysteine (μmol/L) was measured on the kit GenX homocysteine enzymatic method immunoassay using fluorescence polarization immunoassay. A minimum of 8 hours of fasting was required for specimen collection as the amino acid-rich food grossly elevates real homocysteine levels. The measuring range considered was 2.5-50 micromol/L. The normal value of homocysteine level in adults was less than 15 micromoles/L, the moderate level was between 15 and 30 micromol/L, the intermediate level was between 30 and 100 micromol/L, and the severe level was greater than 100 micromol/L. Antenatal women with raised serum homocysteine levels consisting of > 15 micromoles/L were categorized into Group A (Exposed), and antenatal women with normal serum homocysteine levels were categorized into Group B (Unexposed), consisting of participants with levels between 5 and 15 micromoles/L whereas participants with levels < 5 micromol/L were excluded from the study. Antenatal women were followed up monthly till 28 weeks, twice a week till 28-36 weeks, and weekly after 36 weeks till delivery and postpartum for five days. In follow-up visits, blood pressure was monitored at every visit. The data was entered in case record form and entered into a Microsoft Excel sheet (Redmond, USA). 

The outcome measures studied were demographic characteristics, body mass index (BMI), the association of raised serum homocysteine levels, and placenta-mediated complications. The maternal outcomes analyzed were the mode of delivery, indications for cesarean section, and antenatal, intranasal, and postnatal complications. The fetal outcomes studied involved intrauterine growth restriction, intrauterine death, birth weight, and neonatal intensive care unit (NICU) admission.

The data were coded and analyzed in the statistical software STATA version 10.1 (2011). Descriptive statistics were used to summarize continuous variables with a mean and standard deviation. At the same time, categorical variables were summarized by frequency and percentage. Inferential statistics included tests of significance and p-values. The difference in means in the two groups (exposed and unexposed) was assessed by using two independent sample t-tests. The Chi-square test was used to test the association between exposed and adverse outcomes (maternal). A risk analysis appropriate to the design was also performed. Whereby the relative risk (RR) was estimated, along with a 95% confidence interval, for various adverse outcomes (maternal/fetal). A p-value of <0.05 was considered statistically significant.

## Results

A total of 120 antenatal women were involved and were divided into two groups: Group A (exposed) with raised serum homocysteine levels and Group B (unexposed) with normal serum homocysteine levels. The distribution of women according to homocysteine levels is demonstrated in Table 1.

**Table 1:** Distribution of women according to homocysteine levels

Homocysteine level	Interpretation	Frequency	Percentage
5-15	Normal	60	50%
>15	High	60	50%
Total		120	100 %

The age-wise distribution of women demonstrated that in Group A, the maximum number of women were in the age group between 21 and 25 years, consisting of 30 (50.0%) women, followed by 22 (36.7%) between 25 and 30 years, 6 (10%) more than 30 years, and two (3.3%) were less than 20 years. Similarly, in Group B, the maximum number of women were in the age group between 21 and 25 years, consisting of 39 (65.0%) women, followed by 16 (26.7%) between 25 and 30 years, 4 (6.7%) less than 20 years, and six (10%) more than 30 years. 

Additionally, 86.7% of women in Group A had a normal BMI, and 91.6% of women in Group B had a normal BMI. Thus, among both groups, the majority of females had a normal BMI between 18.5 and 24.9 kg/m2. Furthermore, in Group A, the maximum number of patients was 52 (86.7%) who were primigravida, and in Group B, 32 (53.3%) were primigravida.

The distribution of women according to their gestational age is demonstrated in Table 2.

**Table 2:** Distribution according to the gestational age

Gestational age in weeks	High homocysteine level	Percentage	Normal homocysteine level	Percentage
10 weeks	29	48.3 %	28	46.7%
11 weeks	11	18.3 %	10	16.6 %
12 weeks	15	25.0 %	7	11.7 %
13 weeks	5	8.3 %	6	10.0 %
14 weeks	2	3.3 %	9	15.0 %
Total	60	100.0%	60	100.0 %

The other most common associated factor among women with high homocysteine levels was anemia (25%), followed by secondary infertility (15%), hypothyroidism (13.3%), obesity (10%), and hyperthyroidism (5%), and Rh-negative pregnancy (5%). Normal homocysteine levels had hardly any risk factor; only two females had hypothyroidism (3.3%), and one each had obesity, an AS pattern, and anemia with statistically significant results.

The systolic (SBP), diastolic (DBP), and mean arterial blood pressure (MABP) levels among high and normal homocysteine levels are demonstrated in Table 3. There were more incidences of raised blood pressure among women with high homocysteine levels than among women with normal levels, with statistically significant results.

**Table 3:** Blood pressure at high and normal homocysteine levels MABP = Mean arterial blood pressure

Blood pressure	High homocysteine level		Normal homocysteine level	
	Mean	SD	Mean	SD
Systolic	141.6	16.8	116.4	13.8
Diastolic	87.1	10.4	77.5	10.9
MABP	105.3	11.9	90.5	11.4

The distribution of women according to placenta-mediated complications is demonstrated in Table 4.

**Table 4:** Distribution of women based on placenta-mediated complications and risk factors IUGR = Intrauterine growth restriction, IUFD = Intrauterine fetal demise

Placenta mediated complication	High homocysteine level	Percentage	Normal homocysteine level	Percentage	Relative risk	p-value
Abruption	18	30.0 %	2	3.3%	9	0.0002
Severe pre-eclampsia	13	21.66 %	1	1.7 %	13	0.01
IUGR	12	20.0 %	1	1.7 %	12	0.01
Recurrent pregnancy loss (preterm, IUFD, abortions)	4	6.7 %	1	1.7 %	4	0.2
None	13	21.7 %	55	91.6 %	0.2	<0.001
Total	60	100.0 %	60	100.0 %		

**Figure 1:**
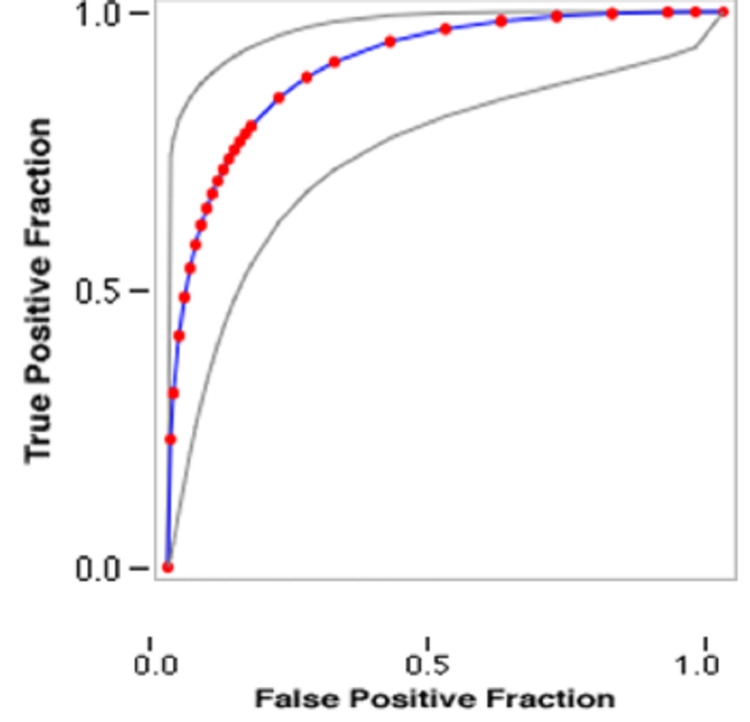
Homocysteine level for placenta-mediated complication as per ROC curve cutoff -45 micromol/L

As per the receiver operating characteristic curve (ROC curve), the cut-off value taken was <5 for low levels of serum homocysteine, 5 to 15 micromol/L for a normal value, and >15 micromol/L for raised serum homocysteine levels. The optimal cut-off value for the present study was 45 micromol/L with a sensitivity of 78.33%, a specificity of 91.67%, a positive predictive value of 90.38%, and a negative predictive value of 80.88% with a diagnostic accuracy of 85%. This means that, for most of the women included in the present study, those who developed placenta-mediated complications had serum homocysteine levels of 45 micromol/L, as shown in Figure 1, or more at 10-14 weeks of gestation.

**Figure 2:**
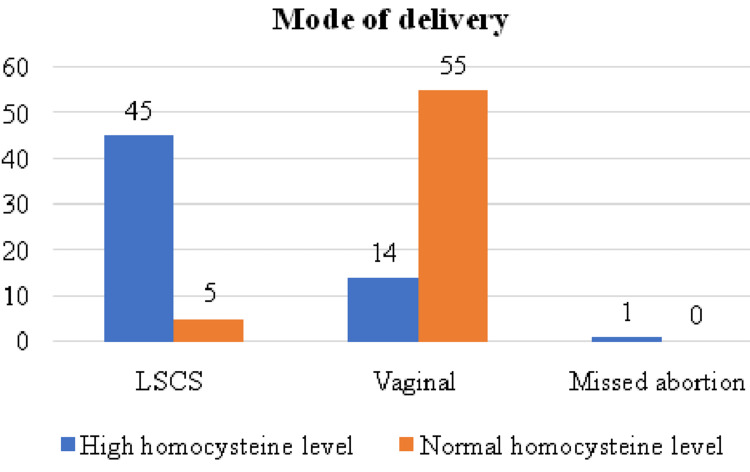
Distribution according to the mode of delivery LSCS = Lower segment caesarean section

In Group A with homocysteine levels > 15 micromol/L, 18 patients (30%) had some complications, whereas 42 patients (70%) had no complications. Only five women (8.3%) had complications, and 55 women (91%) had uncomplicated outcomes in the normal homocysteine level group. Additionally, among the high homocysteine level group, the majority delivered at term, that is, more than 38 weeks (41.7%), whereas 85% of women with normal homocysteine levels also delivered after 38 weeks. The distribution according to the mode of delivery is illustrated in Figure 2.

The indication for lower segment cesarean section (LSCS) among high homocysteine level groups is described in Table 5.

**Table 5:** Indications for LSCS among high homocysteine-level groups LSCS = Lower segment caesarean section, IUGR = Intrauterine growth restriction, CTG = Cardiotocography, MSL = Meconium-stained liquor, HELLP syndrome = Hemolysis, elevated liver enzymes and low platelets, NST = Non-stress test, PROM = Prolonged rupture of membranes, TOLAC = Trial of labor after previous caesarean, PE = Pre-eclampsia

Indication for C-section	Frequency	Percentage
Second stage arrest	4	8.9%
Abnormal Doppler with IUGR	2	4.4%
Abruption with fetal distress	1	2.2%
Breech with IUGR	1	2.2%
Category 2 CTG	2	4.4%
Category 2 CTG with IUGR	1	2.2%
Category 3 CTG with MSL	1	2.2%
Fetal distress	1	2.2%
Impending eclampsia with HELLP Syndrome	1	2.2%
IUGR with non-reactive NST	1	2.2%
Impending eclampsia	1	2.2%
Maternal request	4	8.9%
Missed abortion	1	2.2%
Placenta previa	4	8.9%
Prolonged PROM with preterm with IUGR	1	2.2%
Preterm with breech	1	2.2%
Previous-scar not willing for TOLAC	1	2.2%
Previous-scar with severe PE	1	2.2%
Previous LSCS	1	2.2%
Previous LSCS in labor	1	2.2%
Previous LSCS not willing for TOLAC	1	2.2%
Previous LSCS with mild pre-eclampsia	1	2.2%
Prolonged PROM with severe IUGR	1	2.2%
PROM with fetal distress	1	2.2%
Sever IUGR with abnormal doppler	1	2.2%
Sever pre-eclampsia	1	2.2%
Severe IUGR with abnormal doppler	1	2.2%
Severe oligo IUGR	3	6.7%
Severe PE with breech in labour	1	2.2%
Severe PE with category-2 CTG	1	2.2%
Severe pre-eclampsia	1	2.2%
Severe pre-eclampsia with abnormal doppler	1	2.2%
Total	45	100.0%

The indication for LSCS at normal homocysteine levels is demonstrated in Table 6.

**Table 6:** Indication for LSCS at a normal homocysteine level S and E = Suctioning and Evacuation done, CPD = Cephalopelvic disproportion, TOLAC = Trial of labor after previous caesarean, IUGR = Intrauterine growth restriction, PE = Pre-eclampsia

Indication for LSCS	Frequency	Percentage
CPD with fetal tachycardia	1	20%
Previous scar not willing for TOLAC	1	20%
S&E done	1	20%
Severe oligo IUGR with fetal distress	1	20%
Severe PE with Abruption	1	20%
Total	5	100%

The distribution of groups according to postpartum complications is demonstrated in Table 7, with statistically significant results.

**Table 7:** Distribution according to the post-partum complications PPH = Postpartum hemorrhage

Post-partum Complications	High homocysteine levels	Percentage	Normal homocysteine levels	Percentage	p-value
PPH	10	16.7 %	3	5.0 %	<0.0001
Puperal pyrexia	8	13.3 %	2	3.3 %	
None	42	70.0 %	55	91.7 %	
Total	60	100.0 %	60	100.0%	

**Figure 3:**
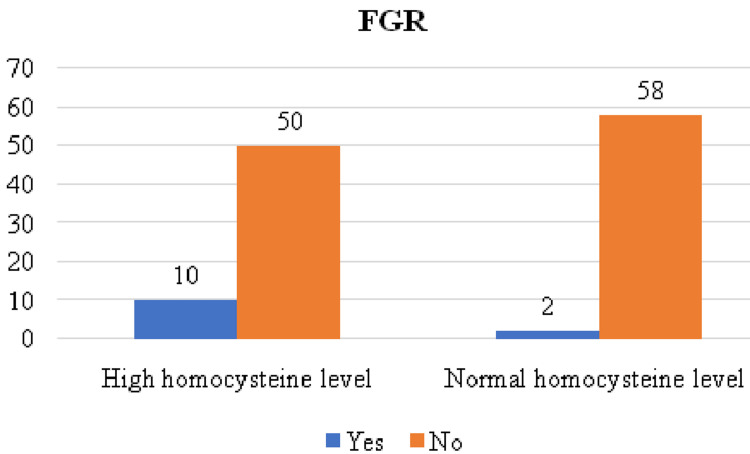
Distribution according to fetal growth restriction FGR = Fetal growth restriction

Among the high homocysteine level group, 10 females had FGR babies (16.7%), and among the normal group, only two had FGR babies (3%), as illustrated in Figure 3.

**Figure 4:**
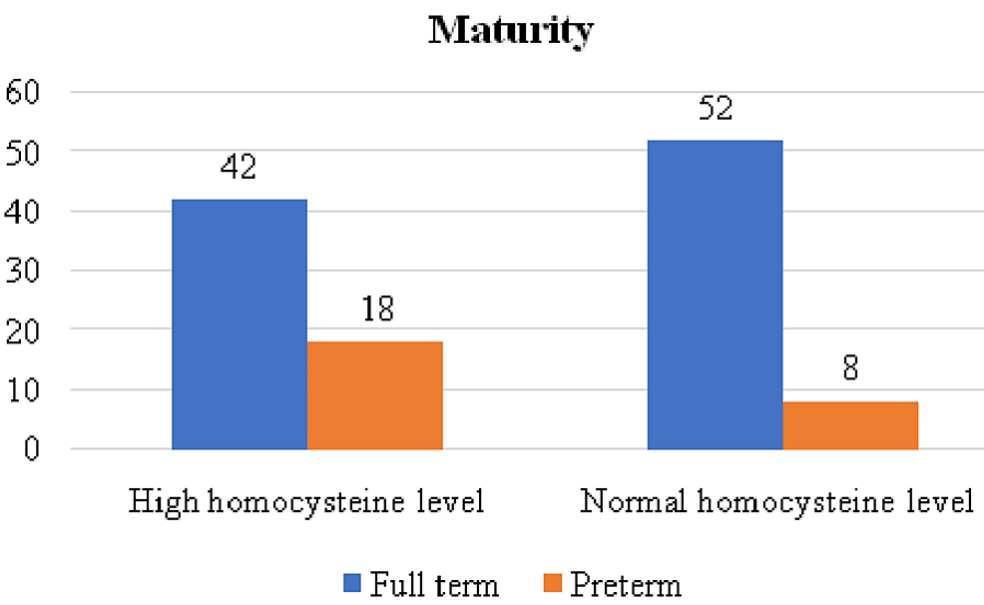
Distribution according to maturity

The distribution according to maturity is depicted in Figure 4.

**Figure 5:**
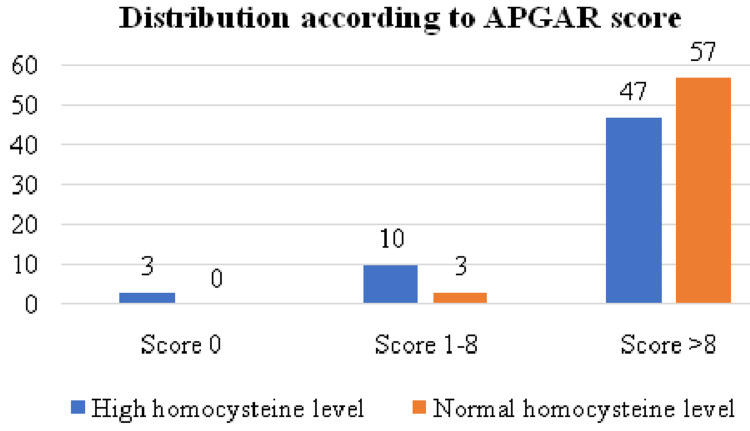
Distribution according to APGAR score

The distribution according to the APGAR score is illustrated in Figure 5.

**Figure 6:**
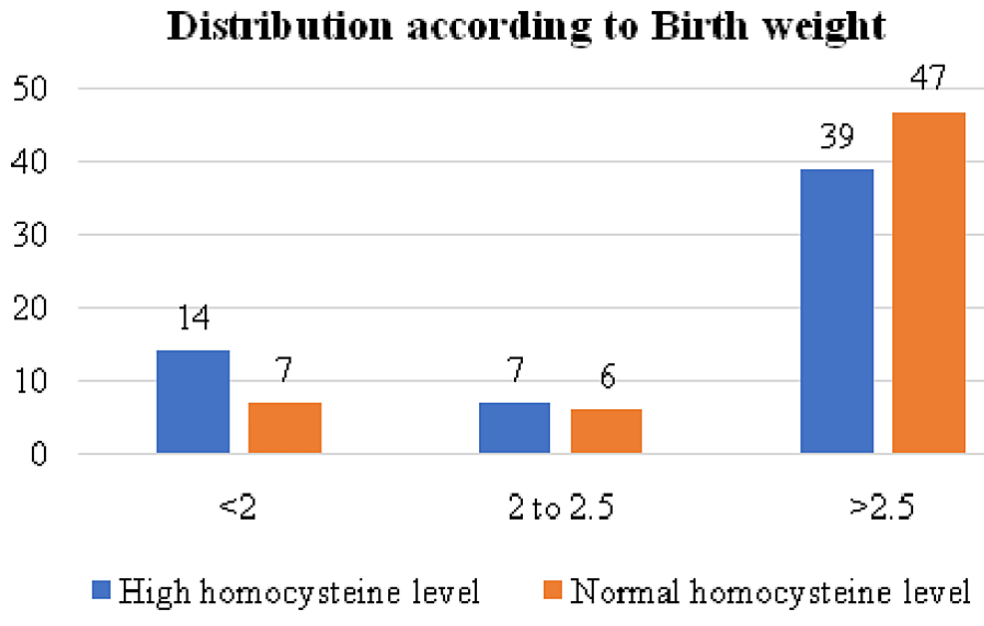
Distribution according to birth weight

The distribution according to birth weight is illustrated in Figure 6. The mean birth weight among the high group was 2.8±0.7 kg, and for the normal group it was 2.9±0.6 kg.

The distribution according to the NICU admission indication is demonstrated in Table 8.

**Table 8:** Distribution according to NICU admission indication NICU = Neonatal intensive care unit, LBW = Low birth weight

NICU admission indication	High homocysteine level	Percentage	Normal homocysteine level	Percentage
No	38	63.0%	52	86.6%
Hyperbilirubenemia	4	6.6%	1	1.6%
LBW	8	13.3%	4	6.6%
Respiratory distress	10	16.6%	3	5%

Among women with high homocysteine levels, 12 (20%) had unsatisfactory fetal outcomes such as NICU admission, preterm birth, and intrauterine demise. Whereas in newborns of women with normal homocysteine, only two (3.3%) had unsatisfactory fetal outcomes.

## Discussion

The present study involved a total of 120 antenatal women. Of them, 4.8% of females had low homocysteine levels, and 47.6% each had normal and high homocysteine levels, respectively. Two groups involving Group A and Group B were formed, in which 60 were exposed to high homocysteine levels, and the other 60 were non-exposed and had normal levels of homocysteine. Ghike et al. described that the total sample size was 60, out of which 17 (28%) women had low levels, 26 (43%) had raised levels, and 17 (28%) had normal homocysteine levels [11]. Similarly, in a study by Chamotra et al., 31% of women had raised values, whereas 68% of women had normal levels of homocysteine [12].

In the present study, among both groups, the majority of females had a normal BMI between 18.5 and 24.9 kg/m2. Chaudhry et al. showed that the mean BMI was 24.9 kg/m2 [4], whereas in the present study, the average BMI for the high homocysteine population was 22.3 kg/m2, and that of the normal population was 21.6 kg/m2. Among the high homocysteine level group, the majority were primigravida, and among the normal group, primi and multigravida were equal. A study by Ghike et al. showed that 18 were primi in group I and 21 were primi in group II [11]. In the present study, in the high homocysteine level group, the majority were primigravida, and women with normal homocysteine levels had an almost equal number of primigravida and multigravida. Similarly, in a study given by Yelikar et al., 50 (41.6%) were primigravida in the high homocysteine level group, and 47.5 (39.5%) were primigravida in the normal homocysteine group [13].

In a study conducted by Gaiday et al. [14], the majority of the population had a mean gestational age of 12 weeks, and Chamotra et al. involved women with 20 weeks of gestation as the study population, whereas in the current study, among both groups, the majority of females had a gestational age of 10 weeks [12]. In a study by Mascarenhas et al., there was no statistical correlation between other associated factors and placenta-mediated complications [15]. In the current study, the most common other associated factors among the high homocysteine level group were anemia, followed by secondary infertility, hypothyroidism, obesity, and Rh -ve pregnancy. Normal homocysteine levels had hardly any other associated factors, with only two females having hypothyroidism and one with obesity, an AS pattern, and anemia. The mean SBP was 117.3±15.6, the mean DBP was 77.7±10.8, the MABP was 90.9±11.9 in the high-level group, and among the normal group, the mean SBP was 116.4±13.8, the mean DBP was 77.5±10.9, and the MABP was 90.5±11.4. Whereas, in a study given by Noori et al., the mean SBP was 155.6, and the mean DBP was 113.3 [16]. Additionally, among high and normal homocysteine level groups, the majority of women delivered after 38 weeks, which correlates well with the study given by Gaidey et al., in which the majority delivered after 38 weeks [14].

Among the high homocysteine level group, the majority of 45 females were delivered by LSCS, followed by 14 by normal vaginal delivery, and one missed abortion was also noted, whereas among normal the homocysteine level group, the majority consisted of 55 females delivered by normal vaginal delivery, and only five (8.3%) women needed CS. A study by Ghike et al. showed that 17 (57%) needed CS as compared to group I, where four needed CS (10.9%) [11]. Whereas Lindblad et al. reported that in the high-level group, 63 (49%) required CS, and in the normal homocysteine group, 41 (32%) required CS [17].

The majority of females (47) had placenta-mediated complications in the high homocysteine level group, and only five females had complications in the normal homocysteine level group. Complications noted among the high homocysteine level group were abruption in 18 women, 13 had severe pre-eclampsia, 12 had IUGR, four had a recurrent pregnancy loss, and 13 had no complications. And complications noted among the normal homocysteine level group were: two had abruption, one had severe pre-eclampsia, one had IUGR, one had a recurrent pregnancy loss, and 55 had no complication. Eighteen females in the high group and five females in the normal group had postpartum complications. A study by Ghike et al. showed that in the normal group, four females had preeclampsia, and in the high group, 26 had preeclampsia [11], whereas a study by Maru et al. showed that 86% had preeclampsia in the high group [18].

Postpartum complications noted among the high homocysteine level group demonstrated that 10 had PPH and eight had puerperal pyrexia. Among normal homocysteine levels, group 3 had PPH, and group 2 had puerperal pyrexia. A study by Maru et al. showed that 20 IUDs were noted in the high group and only two in the normal group. Furthermore, among the high homocysteine level group, 10 females had FGR babies, and among the normal group, only two had FGR babies [18]. A study by Bergen et al. showed that high homocysteine concentrations were associated with an increased risk of SGA and FGR [19], whereas a study by Ghike et al. showed that 14 had FGR [11]. Among the high homocysteine level group, 18 females delivered preterm, and among the normal group, only eight had preterm deliveries. Lindblad et al. reported that in the high-level group, 32 (25%) women delivered preterm, and in the normal homocysteine group, 33 (25.7%) women delivered preterm [17].

Among the high homocysteine level group, 18 females had eventful maternal outcomes, and among the normal group, only five had eventful outcomes. A study by Maru et al. showed that six (6.6%) had uneventful maternal outcomes among the high group [18]. In the current study, among the high homocysteine level group, 12 females had unsatisfactory fetal outcomes, and among the normal group, only two had unsatisfactory fetal outcomes. A study by Maru et al. showed that 38 had unsatisfactory fetal outcomes in the high-level group, and in the normal group, 13 had unsatisfactory fetal outcomes [18]. In contrast to the above studies, Mascarenhas et al. concluded that 98 had an unsatisfactory fetal outcome in the high-level group and two in the normal group [15].

The limitations of the present study were that it was conducted with small sample size and, therefore, to study the parameters in association with raised homocysteine levels. A larger sample size would be beneficial, as a larger sample size generally provides notable benefits in statistical analysis. One of the main benefits is increased statistical power. With a larger sample size, it is more likely that a statistically significant result will be detected, even if the actual effect size is small. A larger sample size can also lead to a more precise estimation of population parameters, such as means and standard deviations. A larger sample size can lead to more accurate predictions and generalizations from the sample to the population. However, it is essential to note that having a large sample size alone is not a guarantee of a good study, and other factors, such as study design and appropriate statistical analysis, should also be considered. Since there was a statistically significant difference in risk factors between the exposure and non-exposure groups, it may constitute a confounding factor, contributing to the increased adverse outcome risk in the exposure group. A sub-group analysis can be conducted with patients matched for risk factors or regression analysis to ascertain whether these factors had any contribution to the final outcome and the extent of this contribution.

## Conclusions

The current study demonstrated a significant association between high homocysteine levels in antenatal women in the late first trimester and placenta-mediated complications, which showed that women with high homocysteine levels in the late first trimester had more placenta-mediated complications, such as abruption, pre-eclampsia, restricted fetal growth, and recurrent pregnancy losses, compared to women with a normal level of homocysteine in the late first trimester. The cut-off value taken was <5 for low levels of serum homocysteine, 5 to 15 micromol/L for a normal value, and >15 micromol/L for raised serum homocysteine levels. The optimal cut-off value for the present study was 45 micromol/L with a sensitivity of 78.33%, a specificity of 91.67%, a positive predictive value of 90.38%, and a negative predictive value of 80.88% with a diagnostic accuracy of 85%. Therefore, measuring blood homocysteine levels in pregnancy may be helpful as a diagnostic test for the early detection of high-risk individuals for placenta-mediated complications.

Additionally, adverse maternal and perinatal outcomes were seen more commonly in women who had high levels of serum homocysteine in contrast to women with normal homocysteine levels, which implies that excessive homocysteine levels in the first trimester of pregnancy may negatively affect placentation, affecting maternal and perinatal outcomes.
